# Biopierces: drug-eluting ear tags for infection prevention in animal tagging

**DOI:** 10.3389/fvets.2025.1696488

**Published:** 2026-02-20

**Authors:** Christopher Cartmell, Emad Naseri, Russell G. Kerr, Daniel Hurnik, Chelsea Martin, Ali Ahmadi

**Affiliations:** 1 Department of Pharmacology, College of Medicine, Comprehensive Center for Pain and Addiction, Center for Applied Nano Biosciences and Medicine, University of Arizona, Tucson, AZ, United States; 2 Faculty of Sustainable Design Engineering, University of Prince Edward Island, Charlottetown, PE, Canada; 3 Department of Chemistry, University of Prince Edward Island, Charlottetown, PE, Canada; 4 Atlantic Veterinary College, University of Prince Edward Island, Charlottetown, PE, Canada; 5 Department of Mechanical Engineering, École de Technologie Supérieure, Montreal, QC, Canada; 6 University of Montreal Hospital Research Centre (CRCHUM), Montreal, QC, Canada

**Keywords:** animal tagging, biomaterials, drug eluting constructs, infection, piercing

## Abstract

Ear tagging is a routine practice in livestock management, but it can be associated with bacterial colonization and infection at puncture sites. This study evaluated drug-eluting ear tags (Biopierce), incorporating chlorhexidine (CHX) in a poly(lactic-co-glycolic acid) (PLGA) matrix, due to their ability to reduce microbial burden and support wound healing. Biopierce eartags were fabricated by coating commercial ear tags with CHX–PLGA and compared to untreated controls. *In vitro*, Biopierces demonstrated a rapid burst release of CHX (~75% within 2 h), plateauing by 8 h, with eluates showing strong antimicrobial activity against *Staphylococcus aureus* in disk and tag diffusion assays. *In vivo*, five adult commercial boars each received one Biopierce and one control tag, with bacterial colonization assessed at 3, 7, 14, and 28 days using MALDI-TOF identification and semi-quantitative scoring. The Biopierce tags significantly reduced bacterial load, halving the prevalence of heavy contamination (27% vs. 12.6%, *p* = 0.0015) and doubling the prevalence of scant growth (9% vs. 21%, *p* = 0.017). Mean bacterial load scores were significantly lower with Biopierces (2.25 vs. 2.73, *p* < 0.05), and regression modeling confirmed a 20.1% reduction (*p* < 0.001). Histopathology on Day 28 showed trends toward reduced swelling (+45.2% vs. +57.6%) and increased full epithelialization (66% vs. 37%), though these did not reach statistical significance due to the small sample size. Taken together, these results demonstrate that Biopierce eartags provide localized CHX delivery that reduces bacterial colonization at tagging sites and may promote improved healing, supporting their potential as a practical infection and inflammation prevention strategy in livestock management.

## 1 Introduction

Ear tagging is a routine method for livestock identification, playing a central role in traceability programs, herd management, and compliance with national livestock monitoring regulations (1). Despite these advantages, ear tagging is not without complications: the puncture wound created during tag placement represents a direct breach of the skin barrier and a potential nidus for bacterial colonization. In addition, mechanical irritation and tag disturbance also occur from the animal rubbing the tag on objects or other animals, leading to further irritation. Tag-associated infections are of particular concern in swine and cattle, where compromised wound healing can lead to inflammation and pain, decreased feed intake, reduced growth performance, and, in some cases, systemic illness. Reports suggest that infection rates following ear tagging may range from 10 to 30% in commercial operations, with *Staphylococcus aureus*, *Streptococcus* spp., and opportunistic Gram-negative bacteria frequently isolated at the wound site (2). In calves, only 49% of tag wounds were fully healed by 8–11 weeks post-application, underscoring the persistence of tissue damage and the potential for long-term animal welfare implications (3). These issues also carry economic weight, as infection-related losses can reduce productivity and increase veterinary costs.

Conventional strategies to prevent tagging-associated infections have centered on basic hygiene, topical antiseptic sprays, or antibiotics administered at the time of tagging (4). While these approaches can reduce initial bacterial burden, their protective effect is short-lived. Antiseptics are rapidly cleared from the wound site by tissue fluids, and repeated application is impractical in commercial farm settings. Furthermore, systemic or prophylactic antibiotic use is increasingly discouraged due to concerns over antimicrobial resistance, leaving few effective alternatives for sustainable infection control (5). Attempts to redesign ear tags to minimize tissue trauma have reduced mechanical irritation but have not eliminated the fundamental risk of bacterial ingress along the tag post. Thus, there remains an unmet need for interventions that provide sustained, localized antimicrobial activity directly at the tag–tissue interface.

In other biomedical fields, localized antimicrobial delivery has been widely studied as a strategy to prevent device-associated infections. Chlorhexidine (CHX), a broad-spectrum antiseptic with potent activity against both Gram-positive and Gram-negative bacteria, is commonly used in dentistry, orthopedics, and catheter care. Incorporating CHX into biodegradable polymers, such as poly(lactic-co-glycolic acid) (PLGA), allows for sustained release and prolonged antimicrobial activity. For example, CHX-loaded PLGA nanoparticles reduced bacterial adhesion and biofilm formation in dental materials (6), while CHX-eluting coatings on orthopedic fixation devices significantly decreased bacterial colonization *in vivo* (7). Recent advances further underscore the versatility of PLGA-based drug delivery systems for antimicrobial release and tissue healing (8–13). These findings provide a strong rationale for translating CHX–PLGA delivery systems into veterinary applications.

Despite this progress, no previous study has developed or evaluated drug-eluting ear tags for livestock. This represents a critical knowledge gap at the intersection of biomaterials, veterinary medicine, and animal production. The present study addresses this gap by developing Biopierces, commercial ear tags coated with a CHX–PLGA formulation, and evaluating their performance *in vitro* and *in vivo*. We hypothesized that Biopierces would provide rapid yet sustained release of CHX, reduce bacterial colonization at the tag–tissue interface, and support improved wound healing compared to untreated control tags.

## 2 Materials and methods

### 2.1 Fabrication of Biopierces

Chlorhexidine (CHX) was dissolved in dimethyl sulfoxide (DMSO) (Fisher Scientific, USA) to prepare a stock solution. Poly(lactic-co-glycolic acid) (PLGA; lactide:glycolide ratio 85:15, MW = 100–200 kDa; PolySciTech, Indiana, USA) was added to the CHX–DMSO solution to obtain a 10% (w/v) polymer solution. Two formulations were prepared: CHX-50 and CHX-100, corresponding to 50 and 100% (w/w) CHX incorporated into the PLGA matrix, respectively. Commercial Allflex™ Herd Mark Pigtrace ear tags Canadian Pork Council (14) were dip-coated in the CHX-50 and CHX-100 stock solutions and air-dried for 4 h. The dip-coating was repeated twice more to achieve three successive coating layers per tag. Untreated tags from the same production batch served as controls.

### 2.2 *In vitro* drug release study

Phosphate-buffered saline (PBS) (VWR, Radnor, PA, USA) was used as the dissolution medium with pH = 7.4. Biopierces coated with CHX-50 and CHX-100 were left in 5 mL PBS for 2, 4, 8, 12, and 24 h at 37 °C (12, 13, 15), and the medium was stirred at 200 rpm. The medium was dried and resuspended in a 25, 25, and 50% (*v/v*) of DMSO/methanol/PBS solution. A calibration curve was performed, ranging from 1.56 to 100 μg/mL, doubling in increments. At each time period, the solution was subjected to ultra-high performance liquid chromatography high-resolution mass spectrometry, and the results were back-calculated to determine the amount of CHX released at each time point. Liquid chromatography high-resolution mass spectrometry (LC-HRMS) was run for sample integrity analysis and was conducted as follows: Thermo Accela UHPLC Pump, Thermo Exactive HRMS fitted with an ESI source, and Thermo PDA. A kinetex core–shell 100 Å C18 column (2.1 mm × 50 mm, 1.7 μm, Phenomenex) was used with a mobile-phase flow rate of 0.5 mL/min and an injection volume of 10 μL (all samples were prepared in CH_3_OH). The following elution method was used [A = H_2_O (0.1% formic acid), B = CH_3_CN (0.1% formic acid)]: 5% B from 0.0 to 0.2 min, linear gradient from 5% B at 0.2 min to 99% B at 4.8 min, 99% B from 4.8 to 8.0 min, linear gradient from 99% B at 8.0 min to 5% B at 8.5 min, and 5% B from 8.5 to 10.0 min. The following HRMS parameters were used: positive ionization mode, mass resolution of 30,000, mass range of *m*/*z* 190 to 2000, spray voltage of 2.0 kV, the capillary temperature of 300 °C, S-lens RF voltage of 60.0%, maximum injection time of 10 ms, and 1 microscan. The system was controlled by Thermo Xcalibur software modules (12).

### 2.3 *In vitro* CHX efficacy

The efficacy of Biopierces against *S. aureus* was studied in terms of scaffold and disk diffusion assays by characterizing the zone of inhibition of the eluted CHX. *S. aureus* ATCC^®^ 25923TM (Oxoid, Nepean, ON, Canada) is the recommended quality control strain for disk diffusion testing following the Clinical and Laboratory Standards Institute (CLSI) standards (16). The isolate was grown from frozen stock and subcultured twice onto Columbia agar (Oxoid Columbia Blood Agar Base # CM0331) supplemented with 5% defibrinated sheep blood (SBA) (Quad Five Donor Sheep Blood Defibrinated # 610–500) before testing. Colonies were picked from 18 h growth on SBA using a cotton swab, re-suspended in tryptic soy broth (TSB) (BD Bacto Tryptic Soy Broth # 211825), and adjusted to 1.5 × 10^8^ CFU/mL by visual comparison with a 0.5 McFarland standard. A bacterial lawn was inoculated onto cation-adjusted Mueller–Hinton agar plates (MHA) (BD BBL Mueller–Hinton II Agar # 211438). All media were prepared by Atlantic Veterinary College (AVC) Central Services media preparation laboratory following manufacturers’ guidelines.

*Disk diffusion assay.* CHX-100 Biopierces were placed in 5 mL PBS for 2, 4, 8, 12, and 24 h at 37 °C, while stirring at 200 rpm. A total of 30 μL of the dissolution medium was taken and used to moisten sterile paper disks (Whatman, Marlborough, UIK, GE Health Sciences, Chicago, IL, USA) (*d* = 6 mm). The paper discs were dried at room temperature for 2 h and then placed on MHA plates. The plates were incubated for 24 h at 35 ± 2 °C. Images of the MHA plates were taken and analyzed by Fiji image processing software (ImageJ, GNU General Public License) (17). The zone of inhibition was calculated by deducting the disk area from the area of no visible bacterial growth.

*Tag diffusion assay*. The efficacy of the Biopierces was assessed by placing the Biopierces directly on MHA plates. Biopierces coated with CHX-100 solution were used for this purpose. To place Biopierces directly on MHA plates, the bottom parts of the tags were removed before coating. Biopierces were placed directly on MHA plates after drying, and the zone of inhibition was measured after 24 h by deducting the disk area from the no-visual bacterial growth area.

### 2.4 *In vivo* animal tests

*Experimental Animals.* Five healthy, 8-month-old, 120-kg intact Duroc, Yorkshire, and Landrace boars from a commercial farm in Prince Edward Island were selected for this study. The sow farm sells weaned pigs and raises its own replacement breeding stock. The five boars chosen were clinically healthy but had to be culled because they were not selected for the breeding program. Since this was a terminal pilot study, the initial number of animals was limited to five, and they were used at an age when they could be culled. One ear tag was placed in each ear so that all pigs had one negative control tag and one Biopierce placed in the other ear. The placement of Biopierces into the right or left ear was randomly allocated. The pigs remained in the same pens on the farm for the duration of the study. Five control tags and five Biopierce-coated tags with CHX-100 solution were randomly chosen and applied to experimental animals. On day 0 of the study, when the boars were 8 months old, each boar had one control and one treated tag in the alternate ear. The tags were applied by the herd veterinarian in the middle of the pinna in a manner that conventional eartags would be applied. A red Allflex™ universal tagger (22,132, Allflex Livestock Intelligence, Canada) was used to apply the tags to the study pigs. A tag marker was used to give each tag a unique identifier, and the amount of antiseptic applied to each tag was recorded. This research was approved by The University of Prince Edward Island’s Animal Care Committee, file number 6009252.

*Treatment and testing regimen.* Five boars were successfully tagged using the Allflex™ tags and tagger. The day the tags were inserted was recorded as day 0, and the pigs were examined and sampled for the bacterial load on days 3, 7, 14, and 28 days post tagging. A sampling of the tag site was made using a standard bacterial swab with transport medium (14211–754, VWR, USA) inserted under the tag to touch the tag post, and was submitted to the Atlantic Veterinary College Regional Diagnostic Services Bacteriology Lab for routine aerobic culture. All animals remained healthy, continued to eat and grow, and grossly had no detrimental effects from ear tags.

*Analytical methods applied to in vivo swine samples*. Swabs were analyzed using a streak plate procedure, where bacterial colonies detected were identified using matrix-assisted laser-desorption ionization-time of flight mass spectrometry (MALDI-TOF), and Bruker MALDI Biotyper^®^ reported as scant, light, moderate, or heavy growth of that bacterial species. The treatment and control identification were blinded to the diagnostic lab. The results were quantified into a bacterial load score using a four-quadrant streak method. The growth of three or more colonies in the third or fourth quadrant is considered heavy; the growth of three or more colonies in the second quadrant is considered moderate; the growth of ten or more colonies in the first quadrant is considered light; and the growth of ten colonies or lesser in the first quadrant is considered scanty. Scant growth was given a score of 1, light growth = 2, moderate growth = 3, and heavy growth = 4. Quantification included all the isolates identified by the MALDI Biotyper^®^ with a reliable identification. Scores were entered into Stata/IC 14.2 for statistical analysis (18, 19). The prevalence of each category of bacterial growth was analyzed using a chi-squared analysis comparing the prevalence rate of bacterial contamination in treated compared to untreated control tags. The treatment effect on the bacterial load score was analyzed using a random-effect regression model controlling for variation due to sampling day and the individual animal as a random effect. Stata/IC 14.2 software was employed using the xtreg function.

*Postmortem analysis.* On day 28 after tag placement and after the final bacterial sampling, the pigs were sedated and humanely euthanized using a captive bolt. Once death was confirmed, a 10 × 10 cm section of the ear containing the tag was removed and placed into 10% buffered formalin and submitted for histologic examination. Four full-thickness ear samples were prepared from each puncture site. Each of the four samples included the edge of the puncture site, as well as adjacent ear tissue extending towards the ear edges (top, bottom, tip, and toward the head). The samples were marked according to their position on the ear, routinely processed, stained, mounted on glass slides, and assessed using a Nikon Eclipse Ni microscope, camera, and NIS-Elements F image capture software. The assessments were blinded to the treatment or control status of the ear samples.

*Ear Thickness Change.* To determine ear thickness, a transparent standard metric ruler was used to calibrate the imaging software, allowing length measurements to be reported in millimeters. Eight low-magnification images were acquired from each puncture site, corresponding to four images immediately adjacent to the puncture (proximal zone), and four images away from the puncture site and in a normal-appearing area of the ear (distal zone). The thickest aspect of the proximal ear (immediately adjacent to the puncture) was measured in the four images, and the thickness of the normal-appearing distal ear was measured in the four images. Using the thickness of the normal-appearing ear to correct for differences in ear thickness between the pigs allows a standardized comparison between individual animals. In some samples, a determination of thickness could not be made due to an artifact in the sample (part of the edge was missing). In some samples, the puncture site was so thick that the edges extended beyond the field of view of the camera. In those cases, the sample thickness was measured directly with a metric ruler.

*Level of Epithelialization.* In a fully healed ear tag puncture site, the edges of the puncture should be covered by epidermis (epithelial cells). Each of the puncture sites was evaluated to determine if the puncture edge was covered by epithelium (four samples per puncture site were evaluated, as described above). Each sample was assigned to one of three categories of epithelialization: Full (the puncture edge was covered in epithelium), none (there was no epithelium on the puncture edge), or partial (the puncture edge was partially covered by epithelium). In some cases, a determination could not be made because the entire edge had evidence of artifact (torn or peeling tissue).

### 2.5 Statistical analysis

The statistical significance of the histology findings (depth and swelling) was tested again using a random effects regression model controlling for variation in the individual pigs. A chi-squared test was used to determine the difference in the rate of epithelialization between control and treated ear tags (Biopierces).

## 3 Results and discussion

### 3.1 *In vitro* drug release and efficacy study

**Figure 1:**
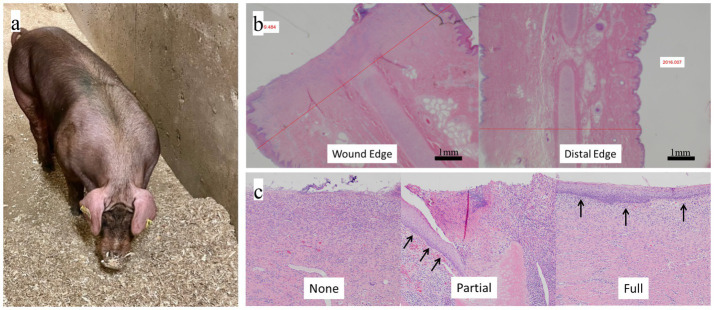
**(a)** Placement of Biopierce and control tags in opposite ears of a pig. **(b)** Histological measurement of ear thickness at wound edge (proximal) versus normal-appearing tissue (distal). **(c)** Representative histological sections showing categories of epithelialization at puncture edges: none, partial (arrows indicate epithelial bridging), and full (arrows indicate continuous epithelial coverage).

*Drug Release Assay*: The release kinetics of CHX-50 and CHX-100 Biopierces over 24 h are shown in Figure 1. Drug quantification was carried out using UHPLC-HRMS, with calibration across concentrations of 1.56 to 100 μg/mL CHX and monitoring of the [M + H^+^] ion at 282.1700 m/z. Biopierces suspended in PBS at 37 °C showed a pronounced burst release within the first 2 h, during which approximately 75% of the drug was eluted. After this phase, release profiles plateaued by 8 h, with no substantial increase through 24 h. For CHX-100 Biopierces, the amount released increased from 43.95 ± 7.90 μg at 2 h to 59.10 ± 13.03 μg at 8 h and stabilized at 59.71 ± 14.37 μg at 24 h. For CHX-50, the release values were 19.82 ± 12.96 μg at 2 h, 36.26 ± 15.84 μg at 8 h, and 31.82 ± 14.79 μg at 24 h. These kinetics are consistent with the burst–plateau profiles reported for PLGA-based release systems, including antimicrobial dressings and scaffolds (6, 7). Comparable results have also been reported for chlorhexidine incorporated into PLGA nanoparticles, where an initial burst release followed by stable antimicrobial activity was observed (20).

Tag Diffusion Assay: Antimicrobial activity was confirmed by placing Biopierces directly on *S. aureus* lawns. Untreated tags and PLGA-only coated controls showed no zones of inhibition, whereas CHX-100 Biopierces produced distinct inhibition zones averaging 411.40 ± 28.14 mm^2^. These results demonstrate that chlorhexidine retained its antibacterial efficacy following incorporation and release from the PLGA matrix. This finding is consistent with earlier PLGA–CHX systems developed for dental applications, where chlorhexidine released from PLGA nanoparticles remained bioactive over extended periods (20).

*Disk Diffusion Assay*: The disk diffusion assay further confirmed the antimicrobial efficacy of Biopierces over time. Sterile paper disks moistened with eluates produced inhibition zones that expanded over the first 12 h and plateaued thereafter, reflecting the drug release profile. For CHX-100, the zone of inhibition increased from 89.29 ± 12.40 mm^2^ at 2 h to 103.84 ± 34.64 mm^2^ at 12 h and plateaued at 101.37 ± 54.25 mm^2^ at 24 h. For CHX-50, the inhibition zone increased from 16.47 ± 9.26 mm^2^ at 2 h to 32.87 ± 6.80 mm^2^ at 12 h and plateaued at 33.88 ± 18.11 mm^2^ at 24 h. The proportional relationship between drug loading and inhibition zone size mirrors the trends observed in other PLGA-based controlled release systems (12).

**Figure 2:**
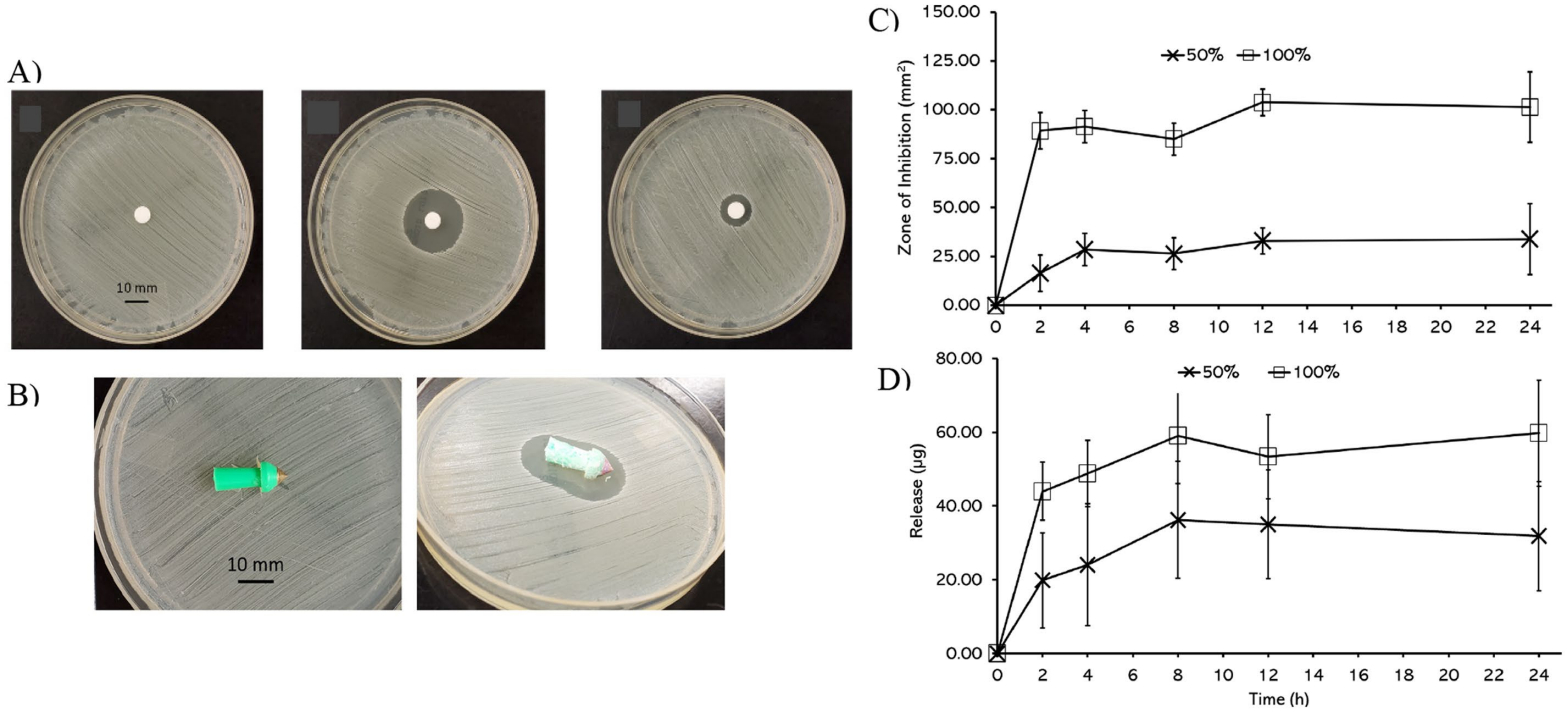
*In vitro* drug release and antimicrobial efficacy of Biopierces. **(A)** Disk diffusion assay: (Deft) negative control, (Middle) positive control, and (Right) CHX-100 Biopierce at the 4 h time point. **(B)** Tag diffusion assay: (Left) untreated control tag and (Right) CHX-100 Biopierce showing an inhibition zone. **(C)** Zone of inhibition areas in disk diffusion assays of CHX-50 and CHX-100 Biopierces over 24 h. **(D)** Cumulative CHX release from CHX-50 and CHX-100 Biopierces measured by UHPLC-HRMS. Error bars indicate standard deviations.

Together, these *in vitro* assays demonstrate that Biopierces provide rapid chlorhexidine release and effective antimicrobial activity against *S. aureus*. The release and efficacy profiles are in agreement with previous PLGA–CHX delivery studies and confirm the suitability of Biopierces for preventing bacterial colonization during the early phase of animal tagging (see Figure 2).

### 3.2 *In vivo* animal test

#### 3.2.1 Bacterial testing

Bacterial colonization outcomes for control and Biopierce-treated ears over the 28-day study are summarized in Table 1. Across 213 isolates (111 from controls and 102 from Biopierces), the distribution of bacterial growth categories shifted significantly in favor of the treated group. Biopierces more than doubled the number of samples showing “scant” growth (21% vs. 9%) while reducing “heavy” contamination by over half (12.6% vs. 27%). The proportions of “light” and “moderate” growth were similar between groups, but the categorical shift away from heavy colonization toward lower-grade growth indicates a consistent antimicrobial effect.

**Table 1:** Summary of bacterial colonization outcomes comparing control and Biopierce-treated tags in pigs over 28 days.

Outcome	Control	Biopierce	*p*-value	Notes
Distribution of bacterial growth (counts, % of total)	Scant: 10 (9%) Light: 40 (36%) Moderate: 31 (28%) Heavy: 30 (27%)	Scant: 21 (21%) Light: 47 (46%) Moderate: 21 (21%) Heavy: 13 (12.6%)	–	Based on 111 control and 102 Biopierce isolates
Prevalence of heavy growth	30/111 (27%)	13/102 (12.6%)	χ^2^ = 10.06 (*p* = 0.0015)	Significant reduction
Prevalence of scant growth	10/111 (9%)	21/102 (21%)	χ^2^ = 5.73 (*p* = 0.017)	Significant increase
Mean bacterial load score	2.73 (95% CI: 2.55–2.91)	2.25 (95% CI: 2.06–2.43)	*p* < 0.05	Lower in Biopierces
Regression model	Reference	−0.54 units vs. control	*p* < 0.001	20.1% reduction after controlling for day & pig

These differences were supported by statistical testing. The prevalence of heavily contaminated samples was significantly higher in controls (χ^2^ = 10.06, *p* = 0.0015), while the prevalence of scant growth was significantly higher in Biopierces (χ^2^ = 5.73, *p* = 0.017). In terms of overall bacterial burden, the mean bacterial load score was lower with Biopierces (2.25, 95% CI: 2.06–2.43) compared to controls (2.73, 95% CI: 2.55–2.91; *p* < 0.05). A regression model accounting for repeated measures and inter-animal variability confirmed that Biopierces reduced the bacterial load score by 0.54 units, corresponding to a 20.1% reduction (*p* < 0.001).

The biological plausibility of these findings is supported by prior research on local chlorhexidine delivery. Chlorhexidine-impregnated dressings for catheter care (21) and orthopedic fixation devices coated with CHX–PLGA composites (22, 23) have similarly demonstrated reduced bacterial colonization at device–tissue interfaces. In dental applications, PLGA nanoparticles carrying chlorhexidine reduced biofilm formation and prolonged antimicrobial activity at adhesive interfaces (20). The current results parallel these reports, showing that Biopierces can leverage the same principle of localized CHX release to suppress colonization in a challenging barn environment.

Importantly, the reduction of “heavy” growth from 27 to 12.6% is not only statistically significant but also clinically meaningful. High-grade colonization has been strongly linked to an increased risk of wound infection and delayed healing in both veterinary and human contexts. By halving the prevalence of heavy contamination and doubling the frequency of low-grade (scant) growth, Biopierces demonstrated a tangible shift in the microbial ecology of the wound–tag interface. The paired-ear design (one treated and one control tag per pig) and blinded microbiological evaluation strengthen the validity of these results despite the small pilot cohort.

Overall, the integrated analysis presented in Table 1 demonstrates that Biopierces significantly reduce bacterial colonization *in vivo*. These findings complement the *in vitro* release and diffusion assays, showing that CHX incorporated into PLGA coatings not only diffuses rapidly but also retains antimicrobial efficacy in real-world conditions. The consistency of these results with published studies on CHX-eluting biomaterials underscores the translational potential of Biopierces for infection prevention in tagging applications and provides a strong rationale for larger animal studies to confirm both microbiological and clinical outcomes.

#### 3.2.2 Pathology

Histopathological outcomes assessing tissue swelling and epithelialization are summarized in Table 2. Ear thickness measurements at the puncture site showed no statistically significant difference between treated and control groups. Mean proximal ear depth was 9.28 mm for Biopierces and 9.85 mm for controls (*p* = 0.19). When thickness was normalized to distal (healthy) tissue, Biopierces again showed numerically lower swelling (+45.2% vs. +57.6%), although this difference was not significant (*p* = 0.21). It is a low number of animals (*N* = 5) in the study that limited the statistical power of the histologic analysis.

**Table 2:** Pathological outcomes comparing Biopierce-treated and control ears.

**Outcome**	**Control**	**Biopierce**	***p*-value**	**Notes**
Ear thickness at puncture site (mm)	9.85 ± 1.62	9.28 ± 0.87	0.19	No significant difference
Relative swelling (% increase vs. distal tissue)	+57.6% ± 7.3	+45.2% ± 7.2	0.21	Biopierces showed less swelling, NS
Epithelialization	None: 4 (21%) Partial: 8 (42%) Full: 7 (37%)	None: 3 (25%) Partial: 1 (8%) Full: 8 (66%)	0.11	Higher full epithelialization with Biopierces

Epithelialization analysis revealed a more favorable healing profile in the Biopierce group. Of the wound edges examined, 66% of treated samples demonstrated full epithelial coverage, compared to only 37% in controls. Conversely, partial epithelialization was much less common in treated samples (8% vs. 42%), while the proportion of wounds showing no epithelialization was similar (25% vs. 21%). Although this difference did not reach statistical significance (*p* = 0.11), the directionality suggests that Biopierces may promote faster or more complete wound closure.

These findings, though based on a limited pilot cohort, are consistent with prior studies demonstrating that local CHX release can improve wound healing outcomes. In dental contexts, chlorhexidine-eluting PLGA nanoparticles reduced inflammation and enhanced re-epithelialization around adhesive interfaces. Similarly, in orthopedic models, CHX-loaded PLGA composites limited swelling and tissue breakdown around fixation devices while supporting tissue repair (22, 23). Although the present pig study did not achieve statistical significance, the observed trends toward reduced swelling and increased epithelial coverage parallel these published outcomes and underscore the potential clinical benefit of CHX-eluting biomaterials.

The lack of significance here likely reflects the small sample size (*n* = 5 pigs), which limited statistical power. Nonetheless, the direction and magnitude of change are biologically meaningful: Biopierces reduced swelling by ~12 percentage points and increased the proportion of fully healed wounds by nearly 30% points compared to controls. In combination with the strong microbiological data (Table 1), these histological outcomes provide convergent evidence that Biopierces not only suppress bacterial colonization but may also support improved healing dynamics at the device–tissue interface.

This proof-of-concept study has several limitations that should be acknowledged. The sample size was small, limited to five animals, and all were male pigs of similar age from a single production setting. As a result, the findings may not fully represent variation across breeds, sexes, environmental conditions, or management systems. In addition, the study was not designed to evaluate clinical infection outcomes beyond colonization burden, and the histological trends we observed did not reach statistical significance due to limited statistical power. Future studies should therefore include larger multi-site studies, comparisons across production environments, and longer-term assessments of wound healing and tag retention.

Despite these limitations, the translational potential of this platform is promising. Beyond ear tagging, similar drug-eluting coatings could be applied to other routine husbandry procedures where localized bacterial ingress contributes to infection and delayed healing. Examples include castration clamps, tail-docking tools, or temporary fixation devices used in identification and neonatal handling (e.g., ear tags in newborn livestock, wing bands in poultry, or umbilical clips in piglets and calves). Such targeted prophylaxis may help reduce early-life infection burden in production animals, support antimicrobial stewardship efforts by decreasing reliance on systemic antibiotics, and improve welfare by maintaining a cleaner wound microenvironment during the critical early healing phase. Moreover, as material formulations and release profiles are further optimized, this platform may also accommodate delivery of other locally acting agents—such as antiseptics, wound-healing modulators, or analgesics—broadening its relevance across species and production settings.

## 4 Conclusion

This study demonstrates that Biopierce-treated ear tags (Biopierces) are capable of delivering chlorhexidine locally and effectively reducing bacterial colonization at tagging sites. *In vitro* assays confirmed both rapid drug release and preserved antimicrobial efficacy against *Staphylococcus aureus*. *In vivo* testing in pigs showed a significant reduction in bacterial load, with a marked decrease in heavily contaminated samples and an increase in low-grade colonization. Histopathological evaluation further suggested favorable trends toward reduced swelling and enhanced epithelialization in Biopierce-treated ears. While this pilot study was performed on a limited number of animals that were only males and of a singular breed, it did show, in a statistically significant manner, that treated tags were able to reduce bacterial contamination of tagged ears. This should be replicated in a larger study with other breeds, sexes, and species.

Taken together, these findings indicate that Biopierces provide both antimicrobial protection and potential support for tissue healing at the device–tissue interface. The results are consistent with prior reports on chlorhexidine-eluting biomaterials used in dental, orthopedic, and catheter applications, underscoring the robustness of this localized delivery strategy. While this pilot study was limited by sample size, the observed effects are biologically and clinically meaningful. Future studies with larger animal cohorts and extended follow-up are warranted to confirm these outcomes and to explore the broader application of Biopierces as a practical intervention for infection prevention in both veterinary and human tagging and implant procedures.

## Data Availability

The original contributions presented in the study are included in the article/supplementary material, further inquiries can be directed to the corresponding author/s.
